# Nationwide Analysis of In-Hospital Mortality in Patients with Encephalitis-Related Diagnoses in Ecuador

**DOI:** 10.3390/diseases14020082

**Published:** 2026-02-21

**Authors:** Karime Montes-Escobar, Christian Eduardo Ramirez-Veloz, Maribel Cecilia Pérez-Pirela, Roy Lincoln Solórzano Giler, Felix Vicente Zambrano Pico, Fanny Soraya Reyes-Mena, Julio Torres, Yulixis Cano, Aline Siteneski

**Affiliations:** 1 Laboratorio de Funcionamiento de Agroecosistemas y Cambio Climático—FAGROCLIM, Departamento de Ciencias Agrícolas, Facultad de Ingeniería Agrícola, Universidad Técnica de Manabí, Portoviejo 130105, Ecuador; karime.montes@gmail.com; 2 Departamento de Formación y Desarrollo Científico en Ingeniería, Facultad de Ingeniería, Ciencia y Tecnología, Universidad Bernardo O’Higgins, Santiago 8370993, Región Metropolitana, Chile; 3 Department of Statistics, University of Salamanca, 37008 Salamanca, Spain; 4 Departamento de Matemáticas y Estadística, Facultad de Ciencias Básicas, Universidad Técnica de Manabí, Portoviejo 130105, Ecuador; cramirez1965@utm.edu.ec (C.E.R.-V.); maribel.perez@utm.edu.ec (M.C.P.-P.); felix.zambrano@utm.edu.ec (F.V.Z.P.); 5 Carrera de Medicina, Facultad de Ciencias de la Salud, Universidad Técnica de Manabí, Portoviejo 130105, Ecuador; rsolorzano0095@utm.edu.ec (R.L.S.G.); fanny.reyes@utm.edu.ec (F.S.R.-M.); 6 Grupo de Investigación de Química Ambiental, Departamento de Química, Facultad de Ciencias Básicas, Universidad Técnica de Manabí, Portoviejo 130105, Ecuador; julio.torres@utm.edu.ec (J.T.); yulixis.cano@utm.edu.ec (Y.C.); 7 Dirección de Investigación, Universidad Técnica de Manabí, Portoviejo 130105, Ecuador

**Keywords:** encephalitis, Ecuador, children, hospitalization, mortality

## Abstract

Background/Objectives: Encephalitis and related acute encephalopathic syndromes represent severe neurological conditions with diverse etiologies and variable clinical outcomes. This study aimed to analyze nationwide hospitalization patterns for encephalitis-related diagnoses in Ecuador between 2018 and 2024. Methods: We used data from the Ecuadorian National Institute of Statistics and Census to estimate age-adjusted hospitalization and mortality rates according to ICD-10 codes. Binary and multinomial logistic regression models were employed to identify sociodemographic factors and diagnostic categories of encephalitis associated with hospitalization and in-hospital mortality. Results: A total of 1560 hospitalizations related to encephalitis-spectrum diagnoses were recorded, with an overall age-adjusted rate of 0.127 per 100,000 inhabitants and 6.0% in-hospital mortality. Unspecified encephalitis and encephalomyelitis were the most common diagnostic categories. Adolescents (10–19 years) were more frequently diagnosed with acute disseminated and bacterial meningoencephalitis, while patients aged ≥70 had higher odds of “other” encephalitis subtypes and the highest mortality risk (aOR = 0.265; 95% CI: 0.116–0.608). Indigenous individuals were more likely to be diagnosed with acute disseminated encephalitis, and Black individuals showed a higher risk for myelopathy associated with human T-cell lymphotropic virus type 1-associated myelopathy. Conclusions: Age and ethnicity significantly influence hospitalization due to encephalitis-related diagnoses in Ecuador. These findings provide epidemiological rates for a lower-middle–income country where the lack of precise diagnosis, age, and ethnicity contribute to the vulnerability of encephalitis.

## 1. Introduction

Encephalitis is an inflammation of the brain parenchyma, often preceded by neurological dysfunction, and infection or autoimmunity [1]. Patients with encephalitis often experience altered consciousness, which can range from mild behavioral abnormalities to deep coma [2]. The most specific symptoms include headache, fever, seizures, altered mental status, focal neurological deficits, cerebrospinal fluid (CSF) pleocytosis, and neuroimaging abnormalities reflecting acute inflammation [3]. Diagnosis of encephalitis requires comprehensive multimodal laboratory testing, neuroimaging, and electrophysiological studies, including bloodwork, BAL/sputum, urine, stool, computed tomography, X-ray, EEG, lumbar puncture, and MRI [4]. Diagnosis is challenging due to variable presentation and the absence of specific biomarkers. Around 50% of cases of autoimmune encephalitis are antibody-negative, and up to 60% of infectious cases lack identifiable pathogens [5,6,7].

Despite a long list of possible microbial etiologies of encephalitis, herpes simplex virus (HSV) is considered the most common sporadic infectious cause [8]. Although viral causes are more common, bacterial, autoimmune, and fungal etiologies can cause encephalitis in both immunocompetent and immunocompromised patients [9]. In addition, non-infectious autoimmune causes of encephalitis have been recognized more frequently in recent years, especially those associated with antibodies against NMDAR, LGI1, and GAD [10]. Geographic region, seasonal and climatic variations, patient age, viral genetic mutations, and changes in immune status over time influence the etiology and epidemiology of encephalitis [11,12,13,14]. Encephalitis occurs more frequently in children, with a pediatric incidence of 10.3 to 13.8 per 100,000 annually, compared with approximately 2.2 per 100,000 in adults [15].

Encephalitis is associated with substantial morbidity and mortality, with the highest incidence observed among pediatric patients and older adults [16]. A Vietnamese study reports an annual hospitalization rate for pediatric encephalitis ranging from 3 to 13 per 100,000 children, with mortality reaching up to 7% [17]. Likewise, an Australian pediatric cohort demonstrated age-specific etiologies of encephalitis, predominated by enteroviruses, parechoviruses, HSV, influenza, *Mycoplasma pneumoniae*, *Streptococcus* spp., ADEM, and anti-NMDAR [18]. Notably, pediatric HSV encephalitis can reach 70% mortality when untreated. Unfortunately, even with appropriate therapy, mortality ranges from 25 to 27%, and approximately 50% of patients experience moderate-to-severe disability, death at 3 months, or develop permanent neurological sequelae [19,20,21]. Finally, apart from infectious causes, immune-mediated encephalitis accounts for up to 34% of pediatric cases and has been associated with increased mortality [22].

There is a lack of data about mortality from encephalitis in the Ecuadorian population. Thus, this study aimed to assess sociodemographic variables and types of encephalitis-hospitalization in Ecuador. The study analyzed hospitalization data over 7 years using information from the National Institute of Statistics and Census. This analysis contributes to robust estimates of mortality from encephalitis in Ecuador. However, these findings help guide public policy objectives in preventing encephalitis among vulnerable groups.

## 2. Materials and Methods

This retrospective cross-sectional study was conducted using hospital records from the National Institute of Statistics and Censuses (INEC) of Ecuador, available at (https://www.ecuadorencifras.gob.ec/camas-y-egresos-hospitalarios/ (accessed on 16 April 2025). The classification used in accordance with the International Statistical Classification of Diseases, 10th Revision (ICD-10). The study population included Ecuadorian patients hospitalized between 2018 and 2024 with a diagnosis of encephalitis. The specific ICD-10 codes considered were: G040 (acute disseminated encephalitis), G041 (myelopathy associated with human T-lymphotropic virus), G042 (bacterial meningoencephalitis and meningomyelitis, not elsewhere classified), G048 (other encephalitis, myelitis, and encephalomyelitis), and G049 (unspecified encephalitis, myelitis, and encephalomyelitis). The study team constructed a database from official records to process and analyze the data. The analysis classified cases according to ICD-10 codes and identified significant associations between sociodemographic variables and mortality.

### 2.1. Variables

The independent variables included the patients’ sociodemographic and contextual characteristics: age groups, sex, ethnicity, type of area of residence (urban or rural), health care sector (public or private), and year of hospital discharge. Additionally, the clinical diagnosis was assessed according to specific ICD-10 codes for encephalitis, and variables were selected for relevance in the epidemiological characterization and their potential influence on clinical outcomes.

The primary dependent variable was the patient’s status at hospital discharge (alive or deceased), considered as a dichotomous clinical outcome reflecting the course and severity of the disease during hospitalization. This variable enables the evaluation of associations between sociodemographic, contextual, and clinical factors, with the aim of identifying risk patterns and potential determinants of mortality due to encephalitis in Ecuador.

### 2.2. Data Processing and Analysis

The data was initially processed and reviewed in Microsoft Excel, removing duplicate records, verifying consistency, standardizing labels, and recoding categorical variables. After validating the dataset, the data were exported to IBM SPSS Statistics version 25 to perform all statistical analyses. The analysis calculated both absolute frequencies and relative proportions and estimated mortality rates across various sociodemographic subgroups. To examine statistical associations between variables such as age, sex, ethnicity, area of residence, and hospital discharge status, the researchers applied Pearson’s chi-square test.

Descriptive analyses were presented as frequencies (*n*) and percentages (%). For the bivariate analysis, Pearson’s chi-square test (χ^2^) or Fisher’s exact test was employed, used to evaluate the association between each sociodemographic variable (rows) and the clinical outcomes or diagnostic subtypes (columns). The *p*-values reported for these tables represent the global significance of the variable. In the multivariate analysis, *p*-values indicate the statistical significance of each specific category as a predictor of mortality relative to a reference group. For the multivariate analysis, a binary logistic regression model was performed to estimate adjusted odds ratios (aORs) and corresponding 95% confidence intervals (95% CI), aimed at identifying sociodemographic factors associated with hospital mortality due to encephalitis (alive = 0; deceased = 1). This methodological approach has been previously applied in studies examining severe infectious diseases in hospital settings.

Additionally, to explore the relationship between sociodemographic variables and specific subtypes of encephalitis hospitalizations, according to the specific ICD-10 for encephalitis, a multinomial logistic regression model was employed. Given the unordered categorical nature of the dependent variable, the “unspecified encephalitis” category was set as the reference group. The results were expressed as adjusted relative risk ratios (aRRRs) with 95% CIs. The selection of variables for the multivariate models was based on data availability in the INEC dataset and supported by prior evidence highlighting the influence of sex, ethnicity, and geographic location on encephalitis-related outcomes.

Finally, crude and age-adjusted hospitalization and hospital mortality rates for encephalitis were calculated. Crude rates were estimated by province and year, while age-adjusted rates were calculated annually, using INEC population projections as denominators. This epidemiological approach, based on age standardization, has been widely implemented in surveillance studies to correct for age distribution effects in infectious diseases. The formulas applied were:

(1) $$Hospitalization\;rate=\left(\frac{number\;of\;cause\;specific\;hospitalizations}{number\;of\;people\;in\;the\;population\;at\;risk}\right)\times 100,000$$

(2) $$In-hospital\;mortality\;rate=\left(\frac{number\;of\;cause\;specific\;in-hospital\;deaths}{number\;of\;people\;in\;the\;population\;at\;risk}\right)\times 100,000$$

Age-adjusted hospitalization and hospital mortality rates for encephalitis were calculated using IBM SPSS Statistics version 25 for Windows. For the estimation of age-adjusted rates, the annual population projections from INEC stratified by age groups were used as denominators, while the standard population recommended by the World Health Organization (WHO), was incorporated into SPSS as the reference for adjustment. All statistical analyses were interpreted under a significance criterion of *p* < 0.05, which was considered statistically significant.

The physiological significance of the *p*-values reported herein lies in their ability to identify host and environmental factors that objectively influence the inflammatory course of encephalitis. A significant *p*-value (*p* < 0.05) identifies biological risk patterns, such as the increased susceptibility of older adults to fatal outcomes, which is linked to the physiological decline of blood–brain barrier integrity and immune response efficiency.

### 2.3. Ethical Considerations

This study used secondary data from public sources, obtained from the official records of the Instituto Nacional de Estadística y Censos (INEC) [23], without including sensitive information or personal identifiers. This secondary data analysis required no individual consent or ethics approval, complying with confidentiality and international standards for responsible use of health research data.

## 3. Results

During the period 2018–2024, a total of 1560 hospitalizations for encephalitis cases in Ecuador, of which 94 cases (6.0%) resulted in in-hospital mortality (Table 1). Sex distribution, males accounted for 54.8% (*n* = 803) and females for 45.2% (*n* = 663), with no statistically significant differences in mortality (*p* = 0.918). Regarding age groups, the highest proportion of hospitalizations occurred among pediatric patients: 0–9 years (96.4%; *n* = 485) and 10–19 years (96.5%; *n* = 303), followed by the 20–29 year group (94.6%; *n* = 176). However, the burden of mortality was disproportionately higher among middle-aged and older adults. Deaths were concentrated in the 40–49-year (10.3%), 60–69-year (14.9%), and ≥70-year (10.3%) age groups. The association between age and in-hospital mortality was statistically significant (*p* = 0.007), indicating that advanced age is a critical risk factor.

Other sociodemographic variables showed no significant associations with mortality: ethnicity (*p* = 0.180), area of residence (*p* = 0.961), hospital sector (*p* = 0.114), and year of hospital discharge (*p* = 0.820). This finding suggests that, in the Ecuadorian context, in-hospital mortality from encephalitis occurs by patient age rather than by structural factors such as place of residence or type of health facility. Although ethnicity was not significantly associated with mortality, the overwhelming representation of the mestizo group within the cohort may have limited the ability to detect differences across ethnic minorities. The lack of variation in outcomes by discharge year may indicate stable hospital care during the study period. However, the stability may reflect diagnostic limitations and reliance on nonspecific clinical categories.

The etiological classification revealed that unspecified encephalitis, myelitis, and encephalomyelitis (G049) were the most frequent, accounting for 56% of cases (866) (Table 2). The G049 classification in the pediatric population, particularly in the 0–9-year age group (48.1%), reflects a high proportion of clinical diagnoses without etiological confirmation. The magnitude of this group is likely related to limitations in laboratory testing and the absence of standardized microbiological confirmation protocols in public hospitals, which can cause these rates. Secondly, other encephalitis, myelitis, and encephalomyelitis (G048) accounted for 354 cases (23%). The G048 category showed a heterogeneous age distribution, with a predominance of adolescents and young adults, and a progressive increase towards the end of the study period (2023–2024).

Bacterial meningoencephalitis and meningomyelitis not classified (G042) totaled 233 cases (15%), observed mainly in adults aged 40–70 years. This trend is consistent with the higher susceptibility of this age group to invasive bacterial infections, often associated with comorbidities or states of immunosuppression. The high G042 concentration in public hospitals (84.5%) further suggests the centralization of severe case management in referral centers. Acute disseminated encephalitis (G040) accounted for 86 cases (6%), predominantly in children and adolescents. Although less frequent, G040 is clinically relevant due to its association with post-infectious or autoimmune processes. The urban area is most prevalent compared to rural areas. Commonly, urban areas are associated with population concentration and greater access to neurologists and advanced diagnostic techniques.

Finally, myelopathy associated with human T-lymphotropic virus type 1 (HTLV-1) (G041) was the least reported, with 21 cases (1.3%). Despite its low frequency, its detection confirms the presence of the retrovirus in specific population groups, underscoring the need for targeted surveillance in at-risk areas. Overall, the five clinical entities showed no statistically significant differences regarding sex, ethnicity, area of residence, type of hospital, or year of discharge (*p* > 0.05). Nevertheless, a marked concentration of hospitalizations was identified in public hospitals (72%) and in urban areas (88%), reflecting a pattern of centralization in the provision of specialized services. The high proportion of cases categorized as “unspecified” poses a challenge for epidemiological surveillance and public health planning. This finding highlights the need to strengthen differential diagnostic capacity by implementing standardized laboratory protocols, which would improve etiological accuracy and enable more effective guidance for therapeutic and preventive strategies.

During the period 2018–2024, the age-adjusted average hospitalization rate for encephalitis was 0.127 per 100,000 inhabitants (Table 3). The trend showed interannual fluctuations, with a maximum in 2024 (0.1740) and a minimum in 2021 (0.0752), the latter coinciding with the ongoing impact of the COVID-19 pandemic on hospital capacity. Among the diagnostic classifications, encephalitis, myelitis, and encephalomyelitis not otherwise specified accounted for the highest rates in most years, reflecting their predominance within the etiological classification. In contrast, myelopathy associated with the human T-lymphotropic virus (HTLV) exhibited the lowest rates, with virtually null values throughout the study period.

The complementary rate analysis (stratum 2) showed patterns consistent with the main findings. Unspecified encephalitis reached its peak in 2022 (0.0077 per 100,000 inhabitants), while other encephalitis forms recorded an increase in 2024 (0.0016 per 100,000 inhabitants). Conversely, HTLV-associated myelopathy remained at zero throughout the study period, reaffirming its minimal epidemiological prevalence. The high burden of cases without confirmed etiology may represent limitations in diagnostic capacity and reliance on broad or non-specific clinical categories. The marked difference between the highest rates (unspecified encephalitis, up to 0.0986) and the lowest (HTLV-associated myelopathy, absent in several years) underscores the heterogeneity of the hospital burden. The rates highlight the importance of strengthening etiological confirmation processes within the health system.

The adjusted binary logistic regression model identified age as the most significant predictor of in-hospital mortality due to encephalitis (Table 4). Taking the 0–9 years group as the reference, adults aged 40–49 years had an adjusted odds ratio (aOR) of 0.305 (95% CI: 0.136–0.686; *p* = 0.004), those aged 60–69 years had an aOR of 0.153 (95% CI: 0.070–0.334; *p* < 0.001), and patients aged ≥70 years had an aOR of 0.265 (95% CI: 0.116–0.608; *p* = 0.002). These results confirm a progressive decline in survival probability with advancing age, reinforcing the findings from the bivariate analysis and underscoring the clinical relevance of older age as a decisive prognostic factor. Regarding sex, no statistically significant differences were observed in hospital mortality (aOR = 1.046; *p* = 0.845). Regarding ethnicity, although Indigenous groups (aOR = 7.987; *p* = 0.117) and Mestizos (aOR = 4.776; *p* = 0.052) showed trends toward higher mortality, these associations did not reach statistical significance. Similarly, area of residence (rural vs. urban; aOR = 0.861; *p* = 0.691) and health sector (public vs. private; aOR = 0.569; *p* = 0.490) were not significantly associated with clinical outcomes.

Regarding the clinical classification of encephalitis, none of the diagnostic categories showed a statistically significant association with hospital mortality. Nonetheless, a higher odds ratio was observed for the group “other encephalitis, myelitis, and encephalomyelitis” (aOR = 1.905; *p* = 0.260), although this did not reach statistical significance. Finally, the analysis of the year of hospital discharge revealed a significant increase in the probability of mortality in 2021 (aOR = 5.644; 95% CI: 1.176–27.092; *p* = 0.031) compared with 2018, the reference year. No other years showed statistically significant differences. This isolated increase may reflect the repercussions of the COVID-19 pandemic on hospital care. Taken together, the results confirm that advanced age is the main prognostic factor for hospital mortality in patients with encephalitis in Ecuador.

The adjusted multinomial logistic regression model identified significant associations between sociodemographic factors and the diagnoses of encephalitis, meningoencephalitis, and myelitis in Ecuador during 2018–2024 (Table 5). Regarding to age, adolescents aged 10–19 years had a lower probability of being diagnosed with acute disseminated encephalitis (aRRR = 0.201; 95% CI: 0.080–0.507; *p* = 0.001) and with bacterial meningoencephalitis not classified elsewhere (aRRR = 0.337; 95% CI: 0.155–0.730; *p* = 0.006), compared to the reference group (0–9 years). In contrast, patients aged 70 years or older showed a strongly significant association with the diagnosis of other encephalitides, myelitis, and encephalomyelitis (aRRR = 234.90; 95% CI: 27.752–1988.252; *p* < 0.001). The increase in susceptibility among older adults may be due to immunosenescence and the coexistence of comorbidities.

Regarding ethnicity, the indigenous population had a significantly higher risk of being diagnosed with acute disseminated encephalitis (aRRR = 12.807; 95% CI: 1.338–122.614; *p* = 0.027), while the Montubio group showed an inverse association with other encephalitides (aRRR = 0.210; 95% CI: 0.095–0.465; *p* < 0.001). The Black population exhibited an elevated risk of myelopathy associated with human T-cell lymphotropic virus (aRRR = 3.93; *p* = 0.001). These results suggest that ethnic differences in diagnoses may influence hospitalization rates and represent patient vulnerability.

Temporal analysis revealed relevant variations. In 2019, the probability of being diagnosed with bacterial meningoencephalitis increased significantly (aRRR = 4.373; 95% CI: 2.117–9.034; *p* < 0.001), as did unspecified encephalitis (aRRR = 4.660; 95% CI: 2.601–8.349; *p* < 0.001). Similarly, significant associations for these categories were observed in 2020 and 2023, whereas in 2022 there was an elevated risk for acute disseminated encephalitis (aRRR = 4.205; 95% CI: 1.780–9.933; *p* = 0.001) and for other encephalitides (aRRR = 3.987; 95% CI: 1.070–14.857; *p* = 0.039). Temporal analysis revealed relevant variations. In 2019, the probability of being diagnosed with bacterial meningoencephalitis increased significantly (aRRR = 4.373; 95% CI: 2.117–9.034; *p* < 0.001), as did unspecified encephalitis (aRRR = 4.660; 95% CI: 2.601–8.349; *p* < 0.001). Similarly, significant associations for these categories were observed in 2020 and 2023, whereas in 2022 there was an elevated risk for acute disseminated encephalitis (aRRR = 4.205; 95% CI: 1.780–9.933; *p* = 0.001) and for other encephalitides (aRRR = 3.987; 95% CI: 1.070–14.857; *p* = 0.039). These results indicate that interannual fluctuations in diagnoses may be due to the impact of the COVID-19 pandemic on hospital care or improved public health services in the countries.

**Figure 1:**
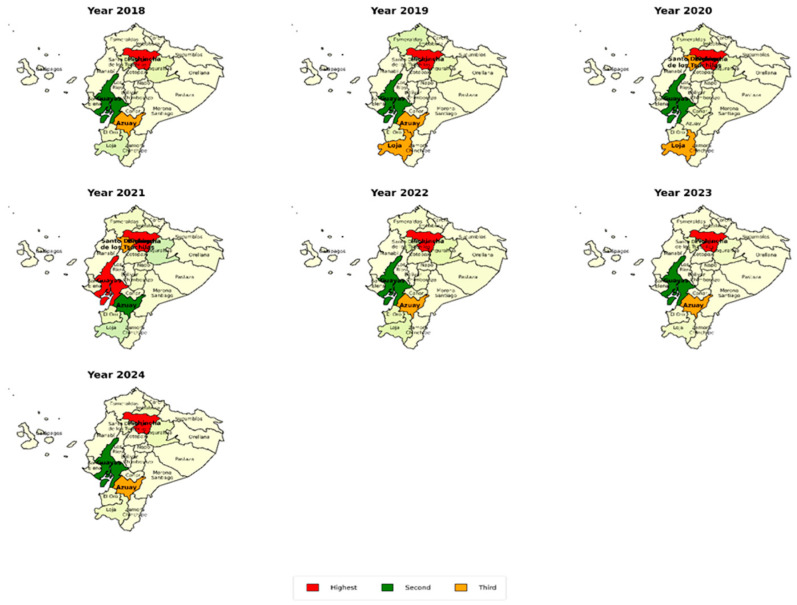
Annual heat maps showing the spatial heterogeneity of encephalitis incidence in Ecuadorian provinces (2018–2024).

Between 2018 and 2024 (Figure 1), spatial analysis revealed a consistent concentration of encephalitis-related hospitalizations in Pichincha, identifying it as the primary national hotspot, particularly in 2019 (37.1%), 2023 (48.9%), and 2024 (42.3%). Guayas ranked second, with notable peaks in 2019, 2022, and 2024, while Azuay frequently ranked third, especially in 2022 (13.3%) and 2024 (12.0%). In isolated years, provinces such as Loja (2019) and Santo Domingo de los Tsáchilas (2023) also registered high burdens. In contrast, Amazonian provinces like Zamora Chinchipe, Bolívar, and Pastaza showed persistently low proportions (<1%). These results suggest the need for surveillance, early diagnosis, and preventive strategies in areas with high encephalitis incidence.

## 4. Discussion

This study reveals that encephalitis-related diagnosis hospitalizations in Ecuador had an age-adjusted average rate of 0.127 per 100,000 inhabitants between 2018 and 2024. The main diagnostic categories for hospitalized patients with encephalitis were unspecified encephalitis and encephalomyelitis not otherwise specified. Adolescents aged 10–19 years represent a more vulnerable group to be diagnosed with acute disseminated or bacterial meningoencephalitis. On the other hand, individuals 70 years or older had markedly elevated risks for “other” encephalitis and myelitis subtypes. Consistently, the mortality model identified advanced age as the strongest predictor of in-hospital death. The indigenous population had a significantly higher risk of being diagnosed with acute disseminated encephalitis. The Black population exhibited an elevated risk of myelopathy associated with human T-cell lymphotropic virus. Our results highlighted ethnic differences in specific encephalitis subtypes for the Ecuadorian population.

There is no clear evidence for a causal association between genetic or ethnic influences and myelopathy associated with human T-cell lymphotropic virus. Previous studies report that adult women represent a vulnerable group [24,25,26]. In Japanese and Caribbean populations, the prevalence of HTLV-1-associated myelopathy is higher in the Caribbean (~1.9%) than in Japan (~0.25%) [27,28]. Our study used CI10 classification and provided high rates of hospitalizations in the Black ethnicity due to human T-cell lymphotropic virus in Ecuadorian patients, which corroborates these ethnic disparities. According to our results, the Indigenous population is a vulnerable population to acute disseminated encephalitis. Then Latin American context confirms that indigenous populations face a higher incidence and mortality from infectious diseases overall [29]. The human herpesvirus 1, known cause of herpes encephalitis, has been previously reported in the Indigenous population from Brazil, with indigenous adults (97.5%) compared to the general population (70.1%) [30]. Although poverty, discrimination, and limited access to healthcare services may contribute to disparities in encephalitis rates [31,32], these factors alone may not fully explain the observed ethnic differences. It highlights the need for future genetic studies to support and clarify epidemiological findings on encephalitis.

In the pediatric population, encephalitis cases are frequently underdiagnosed or misclassified due to nonspecific neurological symptoms and diagnostic barriers [33,34]. In fact, age is a prognostic factor associated with survival and a better prognosis in encephalitis patients [6,35]. In our study, adolescents represented the group at highest risk of hospitalization due to acute disseminated encephalitis or bacterial meningoencephalitis. In contrast with the Chinese population, the highest hospitalization rates for acute encephalitis are more frequent in children under 1 year of age, followed by those aged 1–4 or 5–9 years [16]. Similarly, reports in Spanish hospitalization rates for meningitis are highest in children under 1 year and the 5–9-year age group [36].

Acute disseminated encephalitis or acute disseminated encephalomyelitis and bacterial meningoencephalitis are severe inflammatory disorders of the central nervous system [36]. Both conditions can lead to significant neurological impairment or death if not promptly diagnosed and treated [37]. Acute disseminated encephalomyelitis is most often triggered by viral or bacterial infections, with SARS-CoV-2 recently recognized as a potential cause [38]. Our results show increases in hospital mortality by encephalitis during 2021 equal those of the pandemic period. Mortality rates remained stable in other years but increased with age. It is worth mentioning that no encephalitis subtype showed a statistically significant association with in-hospital mortality. However, the hospitalization rates for those 70 years or older had markedly elevated risks for “other” encephalitis and myelitis subtypes.

Encephalitis affects 12.6 per 10,000 people annually, with mortality rates ranging from 7% to 18% despite current medical advances [39]. Although the national age-adjusted mortality rate remained low during the study period (0.0076 per 100,000 inhabitants), this can reflect the underdiagnosis of in-hospital encephalitis. According to a study conducted in India, a low- to middle-income country, the mortality rate from encephalitis decreased from 35% to 5% between 1978 and 2020 [40]. On the other hand, in high-income countries such as China, Denmark, and Switzerland, the case fatality rates for encephalitis reached 8.05%, 11%, and between 33 and 36%, respectively [41,42,43]. Similarly, high-income European countries, including Sweden and Germany, as well as other regions worldwide, have reported deaths from tick- and squirrel-borne virus encephalitis with a high mortality rate [44,45,46]. To the best of our knowledge, this is the first study to evaluate nationwide rates of encephalitis hospitalization or in-hospital mortality.

### Limitations and Strengths of the Study

Key clinical variables or social factors that could influence encephalitis patient vulnerability and mortality are not available. The cross-sectional design prevents establishing causal relationships between encephalitis incidence and sociodemographic or ethnic factors. The greatest strength of this study is the inclusion of multicenter national data from official records in Ecuador. This study provides nationally representative data about Encephalitis in-hospital mortality and hospitalization. Encephalitis and encephalopathy are known to have different pathophysiological mechanisms [5,47]. In the present study, this limitation reflects the use of ICD-10 discharge codes, which may group these conditions into overlapping categories and do not imply clinical equivalence. This aspect shows the need for cautious interpretation of findings involving these diagnoses and emphasizes that our analysis focuses on encephalitis-related codes in hospitalizations.

An additional limitation of this study is the exclusive inclusion of G04-coded encephalitis cases. Encephalitic syndromes such as viral, bacterial, autoimmune, and paraneoplastic forms (A80–A89, B00–B09, G05) were not considered, as they are inconsistently reported in the national hospital discharge registry. Consequently, while our findings provide a robust population-level estimate for G04-coded encephalitis, they should not be generalized to the full etiological spectrum of encephalitis. It is well known that encephalitis most commonly affects children, with unknown etiology in many cases, and severe clinical outcomes reported in low-, middle-, and high-income countries [48,49,50,51]. The results of this study have implications for understanding hospitalization rates and mortality from Encephalitis in the pediatric population. Furthermore, our results provide guidelines for strengthening equitable public health policies for ethnic minority populations. Finally, the high proportion of cases categorized as “unspecified” poses a challenge for epidemiological surveillance and public health in low- and middle-income countries.

## 5. Conclusions

This nationwide study provides rates of encephalitis-related hospitalizations in Ecuador between 2018 and 2024. The overall age-adjusted hospitalization rate was low, with the majority of cases classified under unspecified encephalitis and encephalomyelitis. Adolescents (10–19 years) showed increased susceptibility to acute disseminated and bacterial meningoencephalitis. On the other hand, individuals aged ≥70 years exhibited a higher likelihood of being diagnosed with other encephalitis subtypes and had the highest in-hospital mortality. Ethnic differences were observed, with Indigenous individuals at greater risk for acute disseminated encephalitis and Black individuals more frequently diagnosed with myelopathy associated with human T-cell lymphotropic virus type 1. These findings underscore the need to enhance diagnostic precision to improve clinical outcomes. Additionally, preventing mortality and improving prognosis in vulnerable groups with encephalitis needs to be a priority in public health policies.

## Figures and Tables

**Table 1:** Characteristics of all hospitalizations for encephalitis in Ecuador during 2018–2024.

Variables	Alive (*n* = 1466)		Dead (*n* = 94)		
	Frequency	Percentage	Frequency	Percentage	*p* Value
Age groups					0.007
0–9 years	485	96.4%	18	3.6%	
10–19 years	303	96.5%	11	3.5%	
20–29 years	176	94.6%	10	5.4%	
30–39 years	118	92.9%	9	7.1%	
40–49 years	105	89.7%	12	10.3%	
50–59 years	89	92.7%	7	7.3%	
60–69 years	86	85.1%	15	14.9%	
≥70 years	104	89.7%	12	10.3%	
Sex					0.918
Male	803	54.8%	52	55.3%	
Female	663	45.2%	42	44.7%	
Ethnicity					0.180
White	10	0.7%	0	0.0%	
Indigenous	35	2.4%	2	2.1%	
Mestizo	1187	81.0%	71	75.5%	
Montubio	10	0.7%	0	0.0%	
Afro-Ecuadorian/Afro-descendant	6	4.0%	3	3.2%	
Black	2	0.1%	0	0.0%	
Mulato/a	3	2.0%	1	1.1%	
Other	213	5.1%	17	18.1%	
Area of residence					0.961
Urban	1292	88.1%	83	88.3%	
Rural	174	11.9%	11	11.7%	
Sector					0.114
Private	423	28.9%	20	21.3%	
Public	1043	78.7%	74	71.1%	
Encephalitis classification					0.010
Acute disseminated encephalitis	81	5.5%	5	5.3%	
Unspecified encephalitis, myelitis and encephalomyelitis	818	55.8%	48	51.1%	
Bacterial meningoencephalitis and meningomyelitis, not elsewhere classified	205	14.0%	28	29.8%	
Myelopathy associated with human T-cell lymphotropic virus	21	1.4%	0	0.0%	
Other encephalitis, myelitis and encephalomyelitis	341	23.3%	13	13.8%	
Year of discharge					
2018	164	11.2%	12	12.8%	0.820
2019	219	14.9%	10	10.6%	
2020	154	10.5%	16	17.0%	
2021	131	8.9%	2	2.1%	
2022	250	17.1%	21	22.3%	
2023	248	16.9%	16	17.0%	
2024	300	20.5%	17	18.1%	

Note: *p*-values reflect the global association test between sociodemographic characteristics (rows) and discharge status or subtypes (columns) using Pearson’s chi-square or Fisher’s exact test.

**Table 2:** Annual diagnosis of encephalitis-related hospitalizations in Ecuador, 2018–2024.

Variables	Acute Disseminated Encephalitis (*n* = 86)		Encephalitis, Myelitis and Encephalomyelitis, Unspecified (*n* = 866)		Bacterial Meningoencephalitis and Meningomyelitis, Not Elsewhere Classified (*n* = 233)		Human T-Cell Lymphotropic Virus-Associated Myelopathy (*n* = 21)		Other Encephalitis, Myelitis and Encephalomyelitis (*n* = 354)		
	Frequency	Percentage	Frequency	Percentage	Frequency	Percentage	Frequency	Percentage	Frequency	Percentage	*p* Value
Age groups											0.001
0–9 years	21	4.2	242	48.1%	86	17.1%	1	0.2%	153	30.4%	
10–19 years	26	8.3%	183	58.3%	33	10.5%	1	0.3%	71	22.6%	
20–29 years	10	5.4%	106	57.0%	31	16.7%	2	1.1%	37	19.9%	
30–39 years	4	3.1%	85	66.9%	13	10.2%	3	2.4%	22	17.3%	
40–49 years	9	7.7%	75	64.1%	12	10.3%	3	2.6%	18	15.4%	
50–59 years	3	3.1%	54	52.5%	17	17.7%	3	3.1%	19	19.8%	
60–69 years	1	1.0%	53	58.6%	17	16.8%	8	7.9%	22	21.8%	
≥70 years	12	10.3%	68	55.5%	24	20.7%	0	0.0%	12	10.3%	
Sex											0.15
Male	46	53.5%	445	51.4%	136	58.4%	10	47.6%	218	61.6%	
Female	40	46.5%	421	48.6%	97	41.6%	11	52.4%	136	38.4%	
Ethnicity											0.010
White	2	2.3%	6	0.7%	0	0.0%	0	0.0%	2	0.6%	
Indigenous	1	1.2%	19	2.2%	11	4.7%	0	0.0%	6	1.7%	
Mestizo	75	87.2%	702	81.1%	194	83.3%	12	57.1%	275	77.7%	
Montubio	0	0.0%	3	0.3%	3	1.3%	0	0.0%	4	1.1%	
Afro-Ecuadorian/Afro-descendant	0	0.0%	4	0.5%	4	1.7%	0	0.0%	1	0.3%	
Black	0	0.0%	1	0.1%	1	0.4%	0	0.0%	0	0.0%	
Mulato/a	1	1.2%	1	0.1%	1	0.4%	0	0.0%	1	0.3%	
Other	7	8.1%	130	15.0%	19	8.2%	9	42.9%	65	18.4%	
Area of residence											0.320
Urban	80	5.8%	776	56.4%	193	14.0%	18	1.3%	308	22.4%	
Rural	6	3.2%	90	48.6%	40	21.6%	3	1.6%	46	24.9%	
Sector											0.001
Private	35	7.9%	310	70.0%	36	8.1%	2	0.5%	60	13.5%	
Public	51	4.6%	556	49.8%	197	17.6%	19	1.7%	294	26.3%	
Year of discharge											0.001
2018	9	5.1%	117	66.5%	33	18.8%	5	2.8%	12	6.8%	
2019	8	3.5%	130	56.8%	50	21.8%	3	1.3%	38	16.6%	
2020	12	7.1%	94	55.3%	22	12.9%	2	1.2%	40	23.5%	
2021	15	11.3%	72	54.1%	16	12.0%	2	1.5%	28	21.1%	
2022	18	6.6%	167	61.6%	36	13.3%	4	1.5%	46	17.0%	
2023	10	3.8%	137	51.9%	34	12.9%	2	0.8%	81	30.7%	
2024	14	4.4%	149	47.0%	42	13.2%	3	0.9%	109	34.4%	

Note: *p*-values reflect the global association test between sociodemographic characteristics (rows) and discharge status or subtypes (columns) using Pearson’s chi-square or Fisher’s exact test.

**Table 3:** Age-adjusted hospitalization and in-hospital mortality rates for encephalitis and its subtypes, per 100,000 population, Ecuador, 2018–2024.

**Years**		**Age-Adjusted Hospitalization Rates**				
	**All Encefalitis Cases**	**Acute Disseminated Encephalitis**	**Encephalitis, Myelitis and Encephalomyelitis, Unspecified**	**Bacterial Meningoencephalitis and Meningomyelitis, Not Elsewhere Classified**	**Human T-Cell Lymphotropic Virus-Associated Myelopathy**	**Other Encephalitis, Myelitis and Encephalomyelitis**
2018	0.1034	$5.3\times 10^{-3}$	0.0687	0.0194	$2.9\times 10^{-3}$	$7.0\times 10^{-3}$
2019	0.1326	$4.6\times 10^{-3}$	0.0753	0.0290	$1.7\times 10^{-3}$	0.0220
2020	0.0971	$6.9\times 10^{-3}$	0.0536	0.0126	$1.1\times 10^{-3}$	0.0228
2021	0.0752	$8.5\times 10^{-3}$	0.0407	$9.0\times 10^{-3}$	$1.1\times 10^{-3}$	0.0158
2022	0.1600	0.0106	0.0986	0.0213	$2.4\times 10^{-3}$	0.0272
2023	0.1449	$5.5\times 10^{-3}$	0.0752	0.0187	$1.1\times 10^{-3}$	0.0445
2024	0.1740	$7.7\times 10^{-3}$	0.0818	0.0231	$1.6\times 10^{-3}$	0.0598
Mean rate between 2018 and 2024	0.127	$7.0\times 10^{-3}$	0.0706	0.0190	$1.7\times 10^{-3}$	0.0284
**Years**	**Age-Adjusted In-Hospital Mortality Rates**					
	**All Encefalitis Cases**	**Acute Disseminated Encephalitis**	**Encephalitis, Myelitis and Encephalomyelitis, Unspecified**	**Bacterial Meningoencephalitis and Meningomyelitis, Not Elsewhere Classified**	**Human T-Cell Lymphotropic Virus-Associated Myelopathy**	**Other Encephalitis, Myelitis and Encephalomyelitis**
2018	$7.0\times 10^{-3}$	0	$5.3\times 10^{-3}$	$6.0\times 10^{-3}$	0	$1.2\times 10^{-3}$
2019	$5.8\times 10^{-3}$	0	$2.9\times 10^{-3}$	$2.9\times 10^{-3}$	0	0
2020	$9.1\times 10^{-3}$	$1.7\times 10^{-3}$	$4.0\;\times 10^{-3}$	$2.3\times 10^{-3}$	0	$1.1\times 10^{-3}$
2021	$1.1\times 10^{-3}$	$6.0\times 10^{-3}$	0	$6.0\times 10^{-3}$	0	0
2022	0.0124	0	$7.7\times 10^{-3}$	$3.0\times 10^{-3}$	0	$1.8\times {\;10}^{-3}$
2023	$8.8\times 10^{-3}$	0	$3.8\times 10^{-3}$	$3.3\times 10^{-3}$	0	$1.6\times 10^{-3}$
2024	$9.3\times 10^{-3}$	$5.0\;\times 10^{-3}$	$3.8\times 10^{-3}$	$3.3\times 10^{-3}$	0	$1.6\times 10^{-3}$
Mean rate between 2018 and 2024	$7.6\times 10^{-3}$	$4.0\times 10^{-3}$	$3.9\times 10^{-3}$	$2.3\times 10^{-3}$	0	$1.0\times 10^{-3}$

Note: Hospitalization and in-hospital mortality rates are expressed per 100,000 population. For improved precision and readability, values below 0.01 are presented in scientific notation (×10^−3^).

**Table 4:** Multivariate binary logistic regression results for in-hospital mortality due to encephalitis.

Variables	aOR (95% CI)	*p* Value
Age groups		
0–9 years	Reference	
10–19 years	1.072 (0.481–2.389)	0.865
20–29 years	0.656 (0.280–1.537)	0.332
30–39 years	0.493(0.205–1.190)	0.116
40–49 years	0.305 (0.136–0.686)	0.004
50–59 years	0.424 (0.165–1.091)	0.075
60–69 years	0.153 (0.70–0.334)	0.000
≥70 years	0.265 (0.116–0.608)	0.002
Sex		
Male	Reference	
Female	1.046 (0.666–1.643)	0.845
Ethnicity		
Afro-Ecuadorian/Afro-descendant	Reference	
White	28,486 (0.00–0.00)	0.999
Indigenous	7.987 (0.595–107.221)	0.117
Mestizo	4.776 (0.988–23.100)	0.052
Montubio	979,596.7 (0.00–0.00)	0.999
Mulato/a	1090 (0.30–39.938)	0.963
Black	1,786,650 (0.00–0.00)	0.999
Other	4.535 (0.876–23.475)	0.072
Area of residence		
Urban	Reference	
Rural	0.861 (0.412–1.181)	0.691
Sector		
Private	Reference	
Public	0.569 (0.324–0.997)	0.49
Encephalitis classification		
Acute disseminated encephalitis	Reference	
Unspecified encephalitis, myelitis and encephalomyelitis	1.20 (0.449–3.212)	0.716
Bacterial meningoencephalitis and meningomyelitis, not elsewhere classified	0.544 (0.190–1.555)	0.256
Myelopathy associated with human T-cell lymphotropic virus	1.691 × 10^16^ (0.00–0.00)	0.997
Other encephalitis, myelitis and encephalomyelitis	1.905 (0.620–5.850)	0.260
Year of discharge		
2018	Reference	
2019	1.984 (0.777–5.065)	0.152
2020	0.688 (0.294–1.610)	0.388
2021	5.644 (1.176–27.092)	0.031
2022	0.861 (0.386–1.916)	0.713
2023	1.096 (0.472–2.545)	0.832
2024	1.577 (0.674–3.689)	0.293

Odds ratios adjusted (aORs) and their 95% confidence intervals (95% CIs) for age group, sex, ethnicity, area of residence, health center sector, encephalitis classification, and year of discharge. Values in bold are statistically significant.

**Table 5:** Multinomial logistic regression of sociodemographic factors associated with encephalitis subtypes hospitalization.

Variables	Acute Disseminated Encephalitis			Encephalitis, Myelitis and Encephalomyelitis, Unspecified			Bacterial Meningoencephalitis and Meningomyelitis, Not Elsewhere Classified		
	aRRR (95% CI)	*p* Value		aRRR (95% CI)		*p* Value	aRRR (95% CI)		*p* Value
Age groups									
0–9 years	Reference			Reference			Reference		
10–19 years	0.201 (0.80–0.507)	0.001		0.423 (0.23–0.802)		0.008	0.337 (0.155–0.730)		0.006
20–29 years	0.486 (1.92–1.234)	0.129		0.563 (0.288–1.101)		0.093	0.249 (0.108–0.574)		0.001
30–39 years	0.330 (0.111–0.977)	0.45		0.616 (0.300–1.266)		0.187	0.361 (0.150–0.868)		0.023
40–49 years	0.311 (0.85–1.137)	0.77		0.977(4.50–2.120)		0.952	0.337 (0.124–0.918)		0.033
50–59 years	0.665 (0.213–2.079)	0.483		0.899 (0.405–1.996)		0.794	0.298 (0.106–0.833)		0.021
60–69 years	0.267 (0.064–1.107)	0.069		0.707 (0.313–1.597)		0.404	0.663 (0.245–1.793)		0.418
≥70 years	0.152 (0.21–1.126)	0.65		0.629 (0.228–1.735)		0.370	0.475 (0.142–1.590)		0.227
Sex									
Male	Reference			Reference			Reference		
Female	0.843 (0.541–1.475)	0.659		0.775 (0.586–1.025)		0.074	0.930 (0.640–1.353)		0.706
Ethnicity									
Afro-Ecuadorian/Afro-descendant	Reference			Reference			Reference		
White	0.628 (0.001–484.515)	0.891		1.151 (0.87–15.235)		0.915	5.490 (0.445–67.728)		0.184
Indigenous	12.807 (1.338–122.614)	0.027		0.562(0.067–4.741)		0.596	0.318 (0.008–12.259)		0.538
Mestizo	1.305 (0.143–11.933)	0.814		1.367 (0.426–4.394)		0.599	1.834 (0.460–7.312)		0.390
Montubio	1.299 (0.550–3.068)	0.551		0.977 (0.662–1.443)		0.909	1.305 (0.716–2.379)		0.385
Mulato/a	0.435 (0.004–53.290)	0.734		0.436 (0.072–2.656)		0.360	1.782 (0.267–11.889)		0.551
Black	30.257 (0.761–1203.665)	0.070		0.426 (0.003–52.494)		0.728	0.903 (0.004–193.528)		0.970
Other	0.9826 (3.396 × 10^−7^–2,008,170.394)	0.980		2.076 (0.006–729.102)		0.807	32.933 (0.133–8181.207)		0.214
Area of residence									
Urban	Reference			Reference			Reference		
Rural	0.699 (0.263–1.855)	0.472		0.996 (6.35–1.563)		0.987	1.595 (0.901–2.824)		0.109
Year of discharge									
2018	Reference			Reference			Reference		
2019	3.280 (1.160–9.278)	0.025		4.660 (2.601–8.349)		0.000	4.373 (2.117–9.034)		0.000
2020	1.422 (0.545–3.710)	0.472		2.309 (1.432–3.723)		0.001	2.978 (1.612–5.501)		0.000
2021	1.922 (0.776–4.759)	0.158		1.752 (1.064–2.886)		0.028	1.215 (0.606–2.437)		0.583
2022	4.205 (1.780–9.933)	0.001		1.574 (0.895–2.768)		0.115	1.153 (0.533–2.494)		0.717
2023	2.136 (0.945–4.829)	0.068		2.192 (1.393–3.449)		0.001	1.739 (0.936–3.230)		0.080
2024	0.975 (0.404–2.351)	0.955		1.380 (0.905–2.105)		0.135	1.389 (0.767–2.513)		0.278
**Variables**	**Human T-Cell Lymphotropic Virus-Associated Myelopathy**				**Other Encephalitis, Myelitis and Encephalomyelitis**				
	**aRRR (95% CI)**		***p* Value**		**aRRR (95% CI)**			***p* Value**	
Age groups									
0–9 years	Reference				Reference				
10–19 years	0.337 (0.155–0.730)		0.006		0.269 (0.031–2.362)			0.236	
20–29 years	0.249 (0.108–0.574)		0.001		0.331 (0.035–3.145)			0.336	
30–39 years	0.361 (0.150–0.868)		0.023		0.632 (0.069–5.790)			0.684	
40–49 years	0.337 (0.124–0.918)		0.033		2.829 (0.328–24.392)			0.344	
50–59 years	0.298 (0.106–0.833)		0.021		1.814 (0.209–15.778)			0.590	
60–69 years	0.663 (0.245–1.793)		0.418		3.168 (0.354–28.368)			0.303	
≥70 years	0.475 (0.142–1.590)		0.227		234.900 (27.752–1988.252)			0.000	
Sex									
Male	Reference				Reference				
Female	0.930 (0.640–1.353)		0.706		6.602 (0.314–1.154)			0.126	
Ethnicity									
Afro-Ecuadorian/Afro-descendant	Reference				Reference				
White	3.93 (0.00–0.00)		1.000		0.040 (0.001–2.366)			0.122	
Indigenous	1.00 (0.09–10.16)		1.000		0.103 (0.001–8.690)			0.315	
Mestizo	287.75 (0.00–0.00)		0.998		0.052 (0.002–1.498)			0.085	
Montubio	287.75 (0.00–0.00)		0.998		0.210 (0.095–0.465)			0.000	
Mulato/a	9.60 (1.10–83.71)		0.041		0.067 (7.926 × 10^−6^–574.298)			0.559	
Black	3.93 (3.93–3.93)		0.001		0.009 (2.986 × 10^−6^–25.885)			0.245	
Other	3.93 (0.00–0.00)		1.000		0.002 (1.049 × 10^−7^–23.123)			0.187	
Area of residence									
Urban	Reference				Reference				
Rural	1.595 (0.901–2.824)		0.109		1.257 (0.484–3.263)			0.638	
Year of discharge									
2018	Reference				Reference				
2019	4.373 (2.117–9.034)		0.000		21.166 (6.548–68.420)			0.000	
2020	2.978 (1.612–5.501)		0.000		2.523 (0.768–8.284)			0.127	
2021	1.215 (0.606–2.437)		0.583		2.237 (0.638–7.844)			0.208	
2022	1.153 (0.533–2.494)		0.717		3.987 (1.070–14.857)			0.039	
2023	1.739 (0.936–3.230)		0.080		4.866 (1.596–14.835)			0.005	
2024	1.389 (0.767–2.513)		0.278		1.461 (0.468–4.562)			0.514	

Relative risk ratios (RRa) and their 95% confidence intervals (95% CI) to assess the risk of encephalitis hospitalization. RRa adjusted for age groups, sex, ethnicity, area of residence and year of discharge. Values in bold are of statistical significance.

## Data Availability

The data analyzed in this study are publicly available on the official website of the Ecuadorian National Institute of Statistics and Censuses: https://www.ecuadorencifras.gob.ec/camas-y-egresos-hospitalarios/ (accessed on 16 April 2025). Curated datasets can be made available upon reasonable request.
